# Oleate Prevents Palmitate-Induced Mitochondrial Dysfunction in Chondrocytes

**DOI:** 10.3389/fphys.2021.670753

**Published:** 2021-06-15

**Authors:** Maria Eugenia Vázquez-Mosquera, Mercedes Fernández-Moreno, Estefanía Cortés-Pereira, Sara Relaño, Andrea Dalmao-Fernández, Paula Ramos-Louro, Alejandro Durán Sotuela, Ignacio Rego-Pérez, Francisco J. Blanco

**Affiliations:** ^1^Unidad de Genómica, Grupo de Investigación en Reumatología (GIR), Instituto de Investigación Biomédica de A Coruña (INIBIC), Complexo Hospitalario Universitario de A Coruña (CHUAC), Universidade da Coruña (UDC), A Coruña, Spain; ^2^Centro de Investigación Biomédica en Red de Bioingeniería, Biomateriales y Nanomedicina (CIBER-BBN), Madrid, Spain; ^3^Grupo de Investigación en Reumatología y Salud, Departamento de Fisioterapia, Medicina y Ciencias Biomédicas, Facultad de Fisioterapia, Universidade da Coruña (UDC), A Coruña, Spain

**Keywords:** osteoarthritis, chondrocyte, metabolism, fatty acids, mitochondria, lipid droplets

## Abstract

The association between obesity and osteoarthritis (OA) in joints not subjected to mechanical overload, together with the relationship between OA and metabolic syndrome, suggests that there are systemic factors related to metabolic disorders that are involved in the metabolic phenotype of OA. The aim of this work is study the effects of palmitate and oleate on cellular metabolism in an “*in vitro*” model of human chondrocytes. The TC28a2 chondrocyte cell line was used to analyze the effect of palmitate and oleate on mitochondrial and glycolytic function, Adenosine triphosphate (ATP) production and lipid droplets accumulation. Palmitate, but not oleate, produces mitochondrial dysfunction observed with a lower coupling efficiency, maximal respiration and spare respiratory capacity. Glycolytic function showed lower rates both glycolytic capacity and glycolytic reserve when cells were incubated with fatty acids (FAs). The production rate of total and mitochondrial ATP showed lower values in chondrocytes incubated with palmitic acid (PA). The formation of lipid droplets increased in FA conditions, being significantly higher when the cells were incubated with oleic acid (OL). These results may help explain, at least in part, the close relationship of metabolic pathologies with OA, as well as help to elucidate some of the factors that can define a metabolic phenotype in OA.

## Introduction

Osteoarthritis (OA) is a chronic progressive disorder that first manifests as a molecular derangement (abnormal tissue metabolism), followed by anatomical and/or physiological disorders characterized by cartilage degradation, bone remodeling, osteophyte formation, joint inflammation, and loss of normal joint function that can culminate in illness (Kraus et al., 2015).

New insights into the pathophysiology of OA have produced a division of the disease into different clinical and structural phenotypes, whose definition allows specific treatments (Mobasheri and Batt, 2016). This phenotype appears in young patients and is associated with faster progress of OA. Systemic factors related to associated metabolic pathologies as well as hormonal imbalances seem to participate in its causes. Although this metabolic phenotype can appear in a generalized way, it is more associated with the hand and the knee (Bijlsma et al., 2011). However, mechanisms of metabolic OA remain unknown. Obesity is one of the most known OA-associated risk factors, mainly explained as an effect of joint overload. However, (i) the close association of other metabolic diseases such as insulin resistance, dyslipidemia and hypertension, and OA (Singh et al., 2002; Sowers et al., 2009; Yoshimura et al., 2011); (ii) higher prevalence of OA in non-obese subjects with a metabolically abnormal phenotype compared with obese subjects without the associated metabolic disease (Lee et al., 2015); and (iii) the positive interaction between obesity and OA in joints not subject to overload, such as OA of hands (Reyes et al., 2016); suggest that systemic factors related to metabolic disorders are involved in this OA subgroup. An increase in plasma fatty acids (FAs) is one of the systemic factors related to the different metabolic pathologies (Baylin et al., 2002; Cusi et al., 2007).

The most abundant FA present in the diet and blood are palmitic acid (PA), a long-chain saturated FA, and oleic acid (OL), a monounsaturated FA (Baylin et al., 2002). Synovial fluid, a plasma ultrafiltrate, provides nutrients necessary for the maintenance of articular cartilage. This fluid contains more FA in OA patients than in healthy subjects (Blanco, 2018). The effects of FA in tissues were reported to differ according to the type of FA. Saturated FA, such as PA, have been described to have highly lipotoxic effects, producing apoptosis in different cell types (Alvarez-Garcia et al., 2014; Law et al., 2018; Ramachandra et al., 2018). The lipotoxic effects of PA have also been studied for monounsaturated FA, such as OL, showing in most cases that OL reverses these effects (Gao et al., 2009; Yuzefovych et al., 2010; Mei et al., 2011).

In a limited oxygen availability conditions, chondrocytes are well adapted to maintain their extracellular matrix synthesis function with minimal nutrient input and low oxygen consumption; this is the reason why these cells mainly use the glycolytic pathway to obtain energy. However, the Adenosine triphosphate (ATP) obtained from mitochondrial respiration is known to significantly contribute to the synthesis of collagen and proteoglycans of the extracellular matrix of articular cartilage (Blanco et al., 2004). Mitochondrial dysfunction, reported in human OA chondrocytes, may affect several pathways that have been implicated in cartilage degradation, including oxidative stress, inflammation, cartilage matrix calcification, and chondrocyte apoptosis (Blanco et al., 2011; Vaamonde-García et al., 2012).

Considering that (i) synovial fluid from OA patients has more FA and that these FA are more lipotoxic than those in healthy subjects (Blanco, 2018), even that (ii) accumulated FA have been detected in OA cartilage OA (Cillero-Pastor et al., 2012), (iii) the suggestion that systemic factors related to metabolic disorders are involved in this OA subgroup; (iv) the importance of the chondrocyte’s metabolic function of the in the maintenance of the extracellular matrix of cartilage; in this study, we used an *in vitro* model of human chondrocytes, to characterize the effects of the two primary FA in blood and the diet (PA and OL) on cellular metabolism.

## Materials and Methods

### Cell Culture and Treatment

The T/C-28a2 chondrocyte cell line (HSS Research Institute, New York, NY, United States) was maintained in Dulbecco’s Modified Eagle Medium (DMEM) (Gibco, Grand Island, NY, United States), supplemented with 10% fetal bovine serum (FBS; Gibco), penicillin (100 U/ml)/streptomycin (100 μg/ml) (Gibco). PA, OL, and PA/OL in a ratio of 1:2 stock solutions were prepared at 5 mM in 10% bovine serum albumin (BSA; Sigma Aldrich, St. Louis, MO, United States), as a vehicle for PA 200 mM and OL 700 mM (Sigma Aldrich) solutions. These solutions were incubated at 55° for 30 min (Cousin et al., 2001). Then, for each condition of FA, the different test concentration solutions were prepared in DMEM without FBS. For the basal condition (BC), DMEM without FBS was used, to which the corresponding BSA percentage was added. The experiments were performed after 12 h of incubation with the FA.

### Cell Viability Assay

Cell viability after the exposure to various concentrations of PA, OL, and PA/OL mix (0.4, 0.7, and 1 mM) was assessed using the MTS assay (Promega). The incubation medium with tested compounds (12 h) was changed to a medium with the MTS reagent at the end of exposure and cells were incubated for additional 3 h at 37°C. The absorbance of dissolved formazan was measured at 490 nm in a microplate reader. Data are displayed as a percentage of cells with BC.

### Quantification of Superoxide Anion (O_2_^–^) Production

After exposure to FAs for 12 h, we measure the production of superoxide anion (O_2_***^–^***); cells were incubated with 4 μg/μl of MITOSOX in Hank’s buffered saline (HBSS) (Thermo Fisher Scientific, Waltham, MA, United States) for 15 min at 37°C and 5% CO_2_ in the dark. The pelleted cells were resuspended to measure fluorescence by flow cytometry (Becton Dickinson, Franklin Lakes, NJ, United States).

### Quantitative Real-Time PCR

Real-time Polymerase Chain Reaction (PCR) was performed with a LightCycler 480-II Instrument (Roche Diagnostics, Risch-Rotkreuz, Switzerland) with TAQMAN Universal Master Mix (Roche). Gene expression was calculated relative to the housekeeping gene (*RPL13A*). Sequence primers, probes, and PCR conditions are shown in Supplementary Table 1.

### Extracellular Flux Analysis

The Seahorse XFp Extracellular Flux Analyzer (Agilent Technologies, Santa Clara, CA, United States) was used to estimate the chondrocyte metabolic stress. Based on the presence of sensors sensitive to the concentration of oxygen and protons, this technique allows to measure simultaneously, in living cells, the two main cellular energy pathways: mitochondrial respiration and glycolysis.

#### Mitochondrial Stress Testing

2 × 10^4^ cells were seeded in XFp Cell Culture Miniplates (Agilent Technologies). Following incubation with the FA, the assay was carried out following the manufacturer’s recommendations. The oxygen consumption rate (OCR) was determined in the presence of specific mitochondrial modulators successively added: 2 μM oligomycin (OLG); 1 μM carbonyl cyanide-p-trifluoromethoxyphenylhydrazone (FCCP); and finally a mixture of rotetone and antimycin A (Rot/AntA) 0.5 μM. Each condition was performed in triplicate and the data obtained (normalized by cell number) was used to calculate the Basal respiration, ATP synthesis, Proton leak, Maximal respiration, Spare respiratory capacity, and the Non-mitochondrial respiration, expressed as OCR (pmol/min)/10^3^ cells; and coupling efficiency as a percentage (Supplementary Table 2a).

#### Glycolysis Stress Testing

2 × 10^4^ cells were seeded in XFp Cell Culture Miniplates. Following incubation with the FA, the cells were incubated in medium without glucose or pyruvate and the extracellular acidification rate (ECAR) was measured. Then, the different modulators were separately injected: 10 mM glucose, 2 μM OLG, and 50 mM 2-deoxyglucose (2-DG). Each condition was performed in triplicate and the data obtained in the test was used to calculate the glycolysis rate and the glycolytic capacity, expressed as ECAR (mpH/min)/10^3^ cells; and glycolytic reserve expressed as a percentage (Supplementary Table 2b).

### Assessment of Energy Balance

#### Contribution of Mitochondrial Oxidative Phosphorylation System and Glycolysis to the Production of ATP

Taking into account that, under normal conditions, the stoichiometry ratio of OCR and proton production rate (PPR) with the ATP production is 5 and 1 respectively (Wu et al., 2014), from the OCR and PPR data obtained from the Seahorse XFp Extracellular Flux Analyzer, the rate of ATP production (pmol/min) due to oxidative phosphorylation system (OXPHOS) and glycolysis was estimated.

#### Quantification of Total and Mitochondrial ATP by Luminescence

To measure intracellular ATP, an ATP bioluminescence assay kit (Roche Applied Science) was used according to the manufacturer’s recommendations. T/C-28a2 chondrocytes were treated with FA before incubation for 2 h with a solution of 156 mM NaCl, 3 mM KCl, 2 mM MgSO_4_, 1.25 mM KH_2_PO_4_, 2 mM CaCl_2_ dihydrate, 20 mM HEPES, and 10 mM glucose for the quantification of total ATP; or 5 mM 2-DG and 5 mM of sodium pyruvate for the quantification of mitochondrial ATP. The ATP levels were normalized to the protein concentration.

### Mitochondrial DNA Copy Number Quantification

To confirm the level of mitochondrial DNA (mtDNA) recovered after incubation with FA, the mtDNA copy number was determined by real time polymerase chain reaction as previously described (Fernández-Moreno et al., 2016). The targeted genes were the mitochondrial 12S ribosomal gene (forward 5′-CCACGGGAAACAGCAGTGAT-3′, reverse 5′-CTATTGACTTGGGTTAATCGTGTGA-3′) and RNAseP (forward: 5′-GCACTGAGCACGTTGAGAGA-3′; reverse: 5′-CCAGTCGAAGAGCTCCAGA-3′). For determining mtDNA copy number, an independent standard curve was generated for each gene (12S-rRNA and RNAseP). The total mtDNA copy number was determined from the Ct values and was extrapolated into the external standard curve. The concentration for each gene was obtained in the analyzed samples. MtDNA copy number values were expressed by the ratio 12S rRNA/RNAseP. To normalize the values between all experiments, we established the mtDNA copy number using the BC condition as 100%.

### Evaluation of Lipid Accumulation

10^4^ cells were seeded in each well of a chamber slide (Thermo Fisher Scientific) and incubated with the different FA conditions described above. After fixation for 10 min with a 4% paraformaldehyde solution, the following two stains were applied: OilRed O (1-[2,5-dimethyl-4-(2,5-dimethylphenylazo)phenylazo]-2-naphthol) staining: for 20 min, then counter stained with hematoxylin. The proportion of positively stained cells was analyzed and quantified using ImageJ, “Image Processing and Analysis in Java” software (National Institutes of Health, Bethesda, MD, United States); LD540 (4,4-difluoro-2,3;5,6-bis(tetramethylene)-4-bora-3a,4a-diaza-s-indacene) staining: for 30 min at a 1:10,000 dilution, then counter stained with Hoechst 33258 solution (Sigma Aldrich). To quantify positivity of LD540 staining were analyzed by flow cytometry (Becton Dickinson).

### Quantification of Intracellular Triglycerides

Following 12 h culture of T/C-28a2 cells with the different FA conditions, the cells were collected to determine their triglyceride content. A suspension of the intracellular content of the cells was obtained by sonication. The samples were then centrifuged at 10,000 × *g* for 10 min at 4°C. For the spectrophotometric quantification of triglycerides (TG) the Glycerol Phosphate Oxidase/Peroxidase method was used (Biosystems, Barcelona, Spain), following the manufacturer’s instructions.

### Statistical Analysis

Appropriate statistical analyses were performed using GraphPad Prism v6 software. Differences between the mean of the groups were determined using unpaired and non-parametric Mann–Whitney *U* test. The results are reported as mean ± SD. A *p*-value less than 0.05 was considered significant.

## Results

### Cell Viability Assay

**FIGURE 1:**
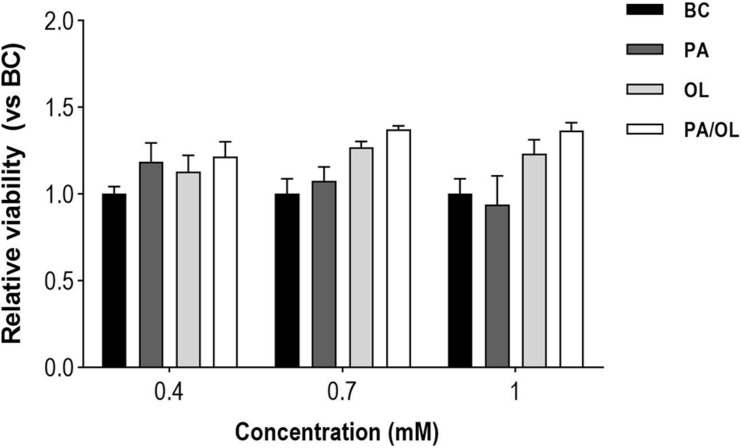
Quantification of the effects of fatty acid concentrations on cell viability. The values, expressed in relation to the basal condition (BC). All data were obtained from three independent experiments, expressed as mean value ± deviation standard.

The effects of different FA concentrations on the chondrocyte cell line T/C-28a2 were evaluated with the CELLTITER 96 Aqueous Non-Radioactive Cell Proliferation Kit Assay. The values obtained for each condition (*n* = 3) were normalized in relation to the BC to which a mean value of 100% was assigned. There are no significant differences between cell viability in BC and that of cells after exposure for 12 h to different concentrations of PA, OL, and PA/OL: 0.4 mM, 0.7 mM, and 1 mM (Figure 1). To investigate whether OL could reverse the possible harmful effects of PA in chondrocytes, we performed viability tests with the co-incubation of monounsaturated and saturated FAs. We included 1:1 and 1:2 saturated/monounsaturated ratios. The viability tests were positive for both combinations at the previously selected concentration (0.7 mM). A 1:2 ratio was chosen for the PA/OL co-incubation because the possible protective effects of OL could thus be more evident and could indicate the impact of an increase in monounsaturated FA in plasma in relation to saturated FA on chondrocytes. From the results obtained in this assay and taking into account the physiological range values of the plasma/serum FA (Joosten et al., 2010; Zhang et al., 2014) we chose 0.7 mM as the concentration to perform the subsequent analyses proposed in this work.

### Palmitate Induces Mitochondrial Dysfunction and Increased Production of Superoxide Anion in Chondrocytes

Globally, cells incubated for 12 h with PA showed a lower OCR (vs. BC). The OCR value was slightly recovered under OL and PA/OL conditions but was always lower than in the BC.

**FIGURE 2:**
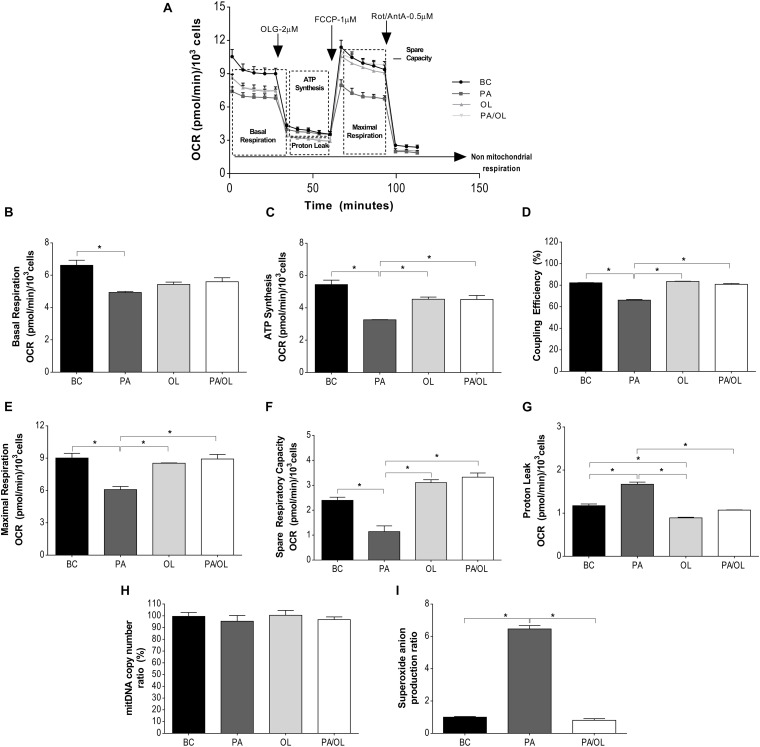
Analysis of mitochondrial function by measurement of the oxygen consumption rate (OCR). **(A)** Representation of the OCR versus time for each of the fatty acid (FA) test solution (without FBS) incubations following the successive addition of each of the modulators of mitochondrial activity: OLG at 2 μM; FCCP at 1 μM and a mixture of Rot/AntA at 0.5 μM. From these OCR values, the following parameters were calculated: **(B)** OCR due to basal respiration; **(C)** ATP synthesis; **(D)** coupling efficiency (%); **(E)** maximal respiration; **(F)** spare respiratory capacity; **(G)** proton leak; **(H)** percentage (%), relative to the basal condition (BC), of the number of copies of mitochondrial DNA; and **(I)** ratio in relation to BC of mitochondrial superoxide anion quantification expression. All data were obtained from three independent experiments, expressed as mean value ± deviation standard; *p < 0.05. BC = basal condition; PA = palmitic acid; OL = oleic acid; OLG = oligomycin; FCCP = carbonyl cyanide-p-trifluoromethoxyphenylhydrazone; ROT = rotenone; AntA = antimycin A. FBS = fetal bovine serum.

Besides, a lower response to respiration modulators was also observed in the PA condition. To assess mitochondrial function more accurately, different parameters that were calculated from the OCR data shown in Figure 2 are explained and analyzed in detail as follows: Cells incubated with FA showed lower basal respiration (Figure 2B) than those in the BC, being statistically significant only after incubation with PA (*p* = 0.034). The OCR corresponding to ATP synthesis (Figure 2C), coupling efficiency (Figure 2D), and maximum mitochondrial respiration (Figure 2E) were significantly lower in cells incubated with PA (*p* = 0.015, 0.001, and 0.030, respectively). In contrast, no significant differences comparing with the BC were found for cells incubated with OL and the PA/OL 1:2 ratio (Figures 2B–E).

Regarding the spare respiratory capacity (Figure 2F), cells incubated with PA showed significantly lower OCR values than the BC (*p* = 0.04), while cells incubated with OL showed higher OCR levels relative to BC, although did not reach the statistical significance (*p* = 0.053).

The OCR due to proton leak (Figure 2G) was significantly higher in cells treated with PA in relation, not only to the BC (*p* = 0.017), but also when compared with OL (*p* = 0.004) and the PA/OL 1:2 ratio (*p* = 0.005). To know if the differences described above could be due to alterations in mitochondrial content, mtDNA was quantified in each of the conditions. As shown in Figure 2H, no significant differences in this parameter were detected. Regarding the levels of mitochondrial superoxide anion (O_2_^–^), these were significantly higher after PA incubation (*p* = 0.002), while no significant difference was detected after PA: OL 1:2 ratio incubation, when compared with the BC (Figure 2I).

### Analysis of Glycolytic Function by Measurement of the ECAR

**FIGURE 3:**
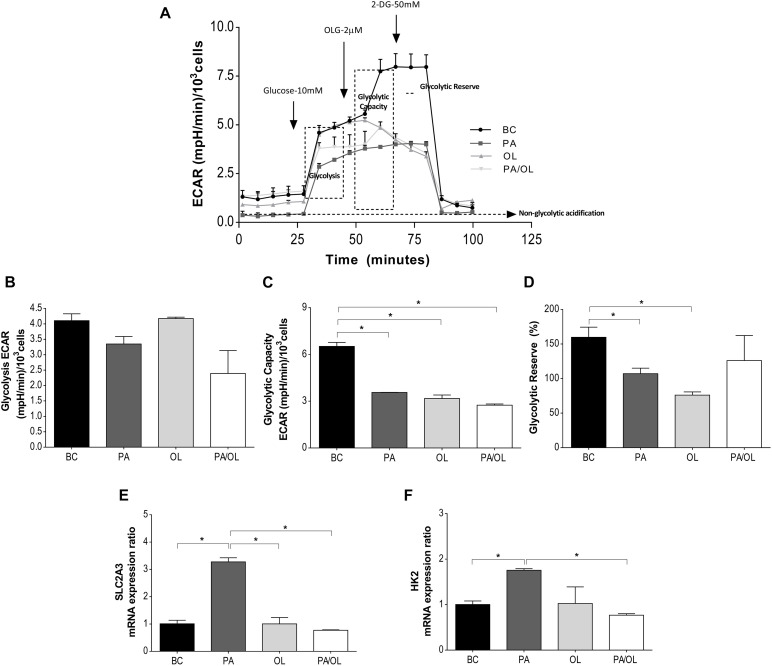
Analysis of glycolytic function by measurement of the rate of extracellular acidification (ECAR). **(A)** Representation of the ECAR versus time for each of the fatty acid (FA) test solution (without FBS)incubations following the addition of glucose at 10 mM, OLG at 2 μM, and 2-DG at 50 μM. From these ECAR values, the following parameters were calculated: **(B)** ECAR due to glycolysis; **(C)** the glycolysis capacity; and **(D)** the glycolytic reserve (%). Gene expression ratios of **(E)** SLC2A3 and **(F)** HK2 after FA incubations are also represented in relation to the BC. All data were obtained from three independent experiments, expressed as mean value ± deviation standard; *p < 0.05. BC = basal condition; PA = palmitic acid; OL = oleic acid; OLG = oligomycin; 2-DG = 2 deoxyglucose; FBS = fetal bovine serum.

To evaluate the main parameters of glycolytic after incubation with the different FA (for 12 h), ECAR data obtained in the SeaHorse XFp Extracellular Flux Analyzer after stimulation with glucose, OLG, and 2-DG, represented in Figure 3A, were analyzed.

The ECAR analysis showed marked differences in each condition in relation to the BC, with those related to the injection of OLG showing the most notable differences (Figure 3A). To define and assess the meaning of the ECAR data, different parameters were calculated. Compared to the BC, a slight, but not significant, decrease in the rate of ECAR due to glycolysis (Figure 3B) was observed in those cells incubated with PA-containing media.

Glycolytic capacity showed significantly decreased rates in cells incubated with PA, OL, and the PA/OL 1:2 ratio (*p* = 0.007, 0.010, and 0.005, respectively) (Figure 3C). In contrast, the glycolytic reserve (Figure 3D) only showed significant reductions in cells incubated with PA (*p* = 0.044) and OL (*p* = 0.015), compared with the BC.

As a complementary analysis of glycolytic function, the gene expression of SLC2A3 and HK2 was quantified. The results showed that incubation with PA-induced significant increases in the expression ratio of both genes (*p* = 0.007 and 0.013, respectively); the expression of these genes, however, was restored to values similar to the BC after incubation with OL and the PA/OL 1:2 ratio (Figures 3E,F).

### Cellular Energy Balance

**FIGURE 4:**
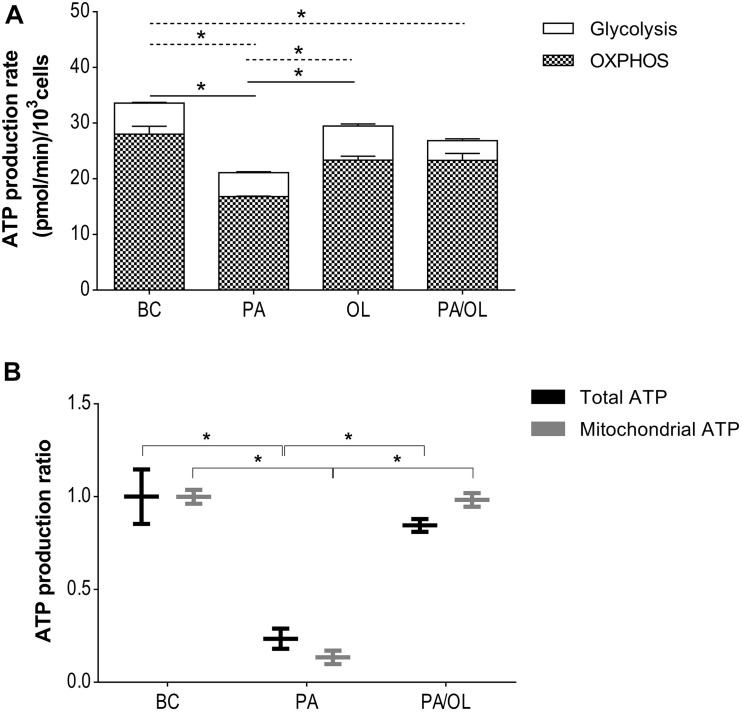
Evaluation of energy balance. **(A)** Total ATP production rate estimated from the values of oxygen consumption (OCR) and the proton production rate (PPR). The production rate of total ATP in pmol/min and the distribution of the rate corresponding to the production by OXPHOS (stripes) and by the glycolytic pathway (white) is represented. Significant differences (*p* < 0.05) are represented by continuous line (OXPHOS) and discontinuous line (glycolytic pathway). **(B)** Measurement of ATP production by luminescence, including both total and mitochondrial ATP. All data were obtained from three independent experiments, expressed as mean ratio in relation to BC ± deviation standard. *p < 0.05. BC = basal condition; PA = palmitic acid; OL = oleic acid; OXPHOS = oxidative phosphorylation.

The metabolic effects observed in the previous experiments should affect cellular energy balance. The OCR and ECAR data obtained were used to estimate ATP produced by OXPHOS and glycolysis. Figure 4A shows the ATP production rates for each of the conditions. The production rate of glycolytic ATP was significantly lower in chondrocytes incubated with PA (*p* = 0.022) and PA/OL (*p* = 0.024) compared to the BC. On the contrary, the production rate of glycolytic ATP was significantly higher in the OL condition when compared to the PA condition (*p* = 0.046). The production rate of ATP from OXPHOS showed lower values in chondrocytes incubated with all of the FA test solutions comparing with the BC, being significantly lower in chondrocytes incubated with PA relative to both the basal (*p* = 0.016) and OL (*p* = 0.011) condition.

The production of total and mitochondrial ATP by luminescence was also quantified in those conditions that showed alterations in the estimation of ATP production described above. The results showed a significant decrease in the production of both total ATP (*p* = 0.040) and mitochondrial ATP (*p* = 0.004) (Figure 4B) in cells incubated with PA compared to BC. In the case of the combined PA/OL incubation, no significant differences in both total and mitochondrial ATP were detected compared to the BC. However, both were significantly higher than the PA (*p* = 0.011 and 0.004, respectively).

### Evaluation of Lipid Accumulation

**FIGURE 5:**
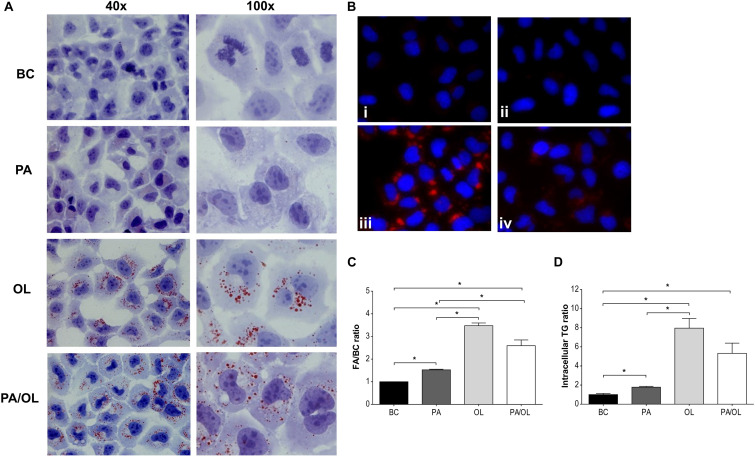
Analysis of lipid droplet formation. **(A)** Images (400×) obtained by optical microscopy with Oil Red O dye and **(B)** by fluorescence microscopy with the LD540 stain, following incubations with the following fatty acid (FA) test solutions (without FBS): (i) BC, (ii) PA, (iii) OL, and (iv) PA/OL. **(C)** Quantification by flow cytometry of the LD540 signal. **(D)** Triglyceride intracellular levels. All data were obtained from three independent experiments, expressed as mean ratio in relation to BC ± deviation standard; *p < 0.05. BC = basal condition; PA = palmitic acid; OL = oleic acid; TG = triglyceride; FBS = fetal bovine serum.

Visualization of prepared images using phase-contrast optical microscopy revealed a considerable accumulation of cytoplasmic structures in cells incubated for 12 h with the different FA test solution sin relation to the BC (Supplementary Figure 1). Therefore, the possible formation of Lipid Droplet (LD) after staining with both OilRed O (Figure 5A) and LD540 (Figure 5B) was evaluated, demonstrating an increase of these structures in FA test solution conditions that were significantly higher in cells incubated with OL (Figure 5C).

LD are structures with a hydrophobic center formed mainly by TG. Therefore, the presence of these glycerols was subsequently quantified in cell extracts obtained after incubation with the different FA test solutions. The analysis of the obtained absorbance values allowed us to determine that TG content is significantly higher after incubation with PA, OL, and PA/OL (*p* = 0.042, 0.021, and 0.026, respectively) when compared with the BC, in agreement with the results obtained from LD stains. Moreover, the TG content of cells incubated with OL is significantly higher than cells incubated with PA (*p* = 0.026) (Figure 5D).

## Discussion

In recent years, the relationship between MS and OA has generated controversy. Different studies performed on the knee and hip joints lose the association after adjusting for body mass index (BMI) (Felson et al., 1988). However, the metabolic alterations that define the MS are considered among the four major environmental factors that are associated with the OA pathogenesis (Berenbaum et al., 2018). Besides, (i) studies on painful interphalangeal OA showed that MS is significantly associated with OA, even adjusting for both age and BMI (Sanchez-Santos et al., 2019) and (ii) a follow-up study carried out on a high number of subjects suggests that both overweight and obesity increase the risk of incident hand OA, a joint not subjected to mechanical overload (Reyes et al., 2016). These findings suggest that there are systemic markers related to metabolic disorders that act as etiological factors of metabolic OA. Among these markers, increased levels of circulating FA stand out. Alterations of glycolysis and mitochondrial pathway in chondrocytes lead to a lowered ATP production, an alteration already described in OA chondrocytes (Maneiro et al., 2003; Ruiz-Romero et al., 2008; Rego-perez et al., 2013).

One of the most sensitive and early parameters in mitochondrial dysfunction is the coupling efficiency between the electron transport chain and OXPHOS (Brand and Nicholls, 2011). The mitochondrial function assay of this study shows a decrease in the rate of OCR for ATP production after incubation with FA, being only significant after exposure to PA. However, it is the incubation with PA that shows a higher rate of proton leak, thus manifesting a lower coupling efficiency. Furthermore, the PA-treated cells showed significant deficiencies in both maximum respiratory capacity and spare respiratory capacity. This would indicate a lower ability of the chondrocytes to respond to energy demand, or how close to their bioenergetic limit they are (Brand and Nicholls, 2011). Knowing the role that oxidative stress plays in the pathogenesis of OA, we wanted to see if PA-induced mitochondrial dysfunction in our experiments could be favoring this process (Henrotin et al., 2003). As our results show, increased production of the mitochondrial superoxide anion accompanies the observed mitochondrial alterations. PA-induced mitochondrial superoxide anion production was observed in other studies performed in different cell types (Lambertucci et al., 2008; Lee et al., 2017). This finding may be due to an elevation of the inner mitochondrial membrane’s potential that leads to an excess in electron donor production. These, transferred to oxygen, become superoxide (Lee et al., 2017). One of the critical sites of mitochondrial superoxide anion production is complex III of the mitochondrial respiratory chain. Assays carried out on muscle cells show that inhibition of this complex partially reduces the formation of superoxide anion induced by PA (Lambertucci et al., 2008).

On the other hand, when chondrocytes were incubated with the same concentration of OL or PA/OL, no significant variations in the parameters of respiration were observed, showing that OL appears to protect chondrocytes from PA-induced mitochondrial dysfunction.

Palmitic acid-induced mitochondrial dysfunction has been reported in a study where the parameters of mitochondrial respiration were analyzed with the same methodology used in our work (Patková et al., 2014). In their paper, the authors detected differences in mitochondrial function according to the type of muscle cell incubated with PA, and they correlated these differences with the amount of mitochondria. In our case, however, the deficient mitochondrial metabolism following the incubation of chondrocytes with PA seems to be independent of the number of mitochondria, as indirectly quantified by calculations of mtDNA copy number by qPCR.

Palmitic acid-induced mitochondrial dysfunction leads to a decrease in the rate of both total and mitochondrial ATP production. This result is in agreement with other studies highlighted in other cell types that analyze damage to mtDNA (Ruiz-Romero et al., 2008; Mei et al., 2011)as well as a decrease in the expression of genes that code for regulatory chain proteins (Hall et al., 2014). In contrast, the ATP production rate is not affected after incubation with OL and partially recovers in PA/OL co-incubation. These results could indicate that saturated FA, such as PA, induce a mitochondrial dysfunction that would lead to an increase in mitochondrial reactive oxygen species, thus favoring apoptosis and inflammation in chondrocytes, a finding previously observed by Alvarez-Garcia et al. (2014).

Studies carried out in pancreatic, renal, or intestinal cells showed alterations in the glycolytic pathway after incubation with PA, including decreases in the expression of both regulatory proteins and this pathway’s activity (Hovsepyan et al., 2010; Botchlett et al., 2016). In our study, the ECAR data showed a lower glycolytic rate after incubation with PA. These observations are in agreement with the studies cited above. In our work, the cellular response when the OXPHOS pathway was inhibited (by adding OLG) showed exciting differences depending on the FA treatment. In the BC, there was an increased ECAR corresponding to an increase in the glycolytic rate to compensate for the mitochondrial pathway’s inhibition. However, when cells were incubated with FAs, the ECAR values showed low glycolytic capacity and reserve. In the presence of PA, a low rate of ATP production was described throughout the glycolytic pathway. In addition to the low rate of ATP production by OXPHOS, this data decreased total ATP production after incubation with PA. Interestingly, the low level of ATP was partially recovered by co-incubation with OL through the recovery of ATP production by OXPHOS.

Despite the low rate of glycolytic activity detected, the results of our study showed an increased expression of SLC2A3 and HK2 after exposure to PA. One possible explanation could be that articular chondrocytes activate mechanisms that favor glucose uptake (SLC2A3) and its integration into the glycolytic pathway (HK2) to compensate for the cellular energy deficit observed both by the lower glycolytic contribution and by mitochondrial dysfunction. In order to confirm this, more studies would be necessary that include, for example, the auxiliary routes of glucose metabolism, such as the pentose phosphate pathway.

The results described thus far reveal harmful effects related to cartilage degradation, on chondrocytes exposed to high levels of PA, which were partially reversed when co-incubated with OL. This protective effect of a monounsaturated FA has also been observed in cells of other peripheral tissues, reversing the effects of PA on insulin resistance, inflammation, mitochondrial dysfunction, oxidative stress, and apoptosis (Coll et al., 2008; Gao et al., 2009; Kadotani et al., 2009; Ricchi et al., 2009; Yuzefovych et al., 2010). Studies carried out in muscle and neuronal cells raise the importance of the expression of factors of mitochondrial biogenesis, the Peroxisome proliferator-activated receptor gamma coactivator 1-alpha (PGC1alpha), and the mitochondrial transcription factor A (TFAM) in the reversal of mitochondrial dysfunction induced by PA (Yuzefovych et al., 2010; Kwon et al., 2014). Another postulate that could explain the protective effect of OL is the cytoprotective capacity of monounsaturated FAs, favoring the formation of TG and their subsequent storage in lipid droplets, as well as the uptake of saturated FAs in these structures (Cnop et al., 2001; Listenberger et al., 2003; Gillet et al., 2015). LDs are considered cellular organellesk of lipid reserve coated with a phospholipid monolayer with multiple associated proteins, and a hydrophobic center composed primarily of TG or cholesterol esters (Walther et al., 2017). In our study, a striking increase in LDs after incubation with the three FA conditions was observed. However, their presence and size were significantly higher when OL was present in the culture medium. These results are supported by the appearance of a higher intracellular TG content after incubation with OL. The difference, both in the amount of LDs and intracellular TG, observed after incubation with PA and the incorporation of OL into the culture medium, may indicate that, as described in other peripheral tissues (Listenberger et al., 2003; Ricchi et al., 2009). In the absence of additional signals, PA has limited ability to be part of TG. The presence of OL promotes the channeling of PA toward the storage of TG, sequestering the PA and thus avoiding its damaging effects on cells. This is supported by the fact that inhibition of TG synthesis leads to the OL acquires the same toxic effects as PA (Listenberger et al., 2003). One possible mechanisms proposed by which OL can promote TG accumulation is that unsaturated FAs can serve as a ligand for transcription factors such as peroxisome proliferator-activated receptor gamma (Kliewer et al., 1997). Although the excessive accumulation of LDs in cells does seem to have long-term lipotoxic effects, it seems that monounsaturated FA, such as OL, offers an initial cytoprotective effect on cells. This issue has not been addressed in our study.

This study has some limitations that must be addressed. On the one hand, the use of primary human chondrocytes or cartilage explants would be an essential support for the results obtained in our work. However, to carry it out, it would be necessary to perform the study, including samples from subjects without OA and no associated metabolic disease, whose availability is limited. This justifies the use in our study of the human chondrocyte cell line T/C-28a2. It is a widely used cell line in *in vitro* studies of OA (Goldring, 2004; D’Adamo et al., 2016; Le Clanche et al., 2018; Nogueira-Recalde et al., 2019). And because it comes from young and healthy cartilage, we consider that it could be the best model to extrapolate the results to chondrocytes of healthy cartilage without associated metabolic disease. On the other hand, an important fact to consider is the possible toxic effect of FA concentrations on cells that could alter the results of tests such as those used in this study. In our case, as in studies conducted on other cell types (Abu Bakar et al., 2017; Chen et al., 2018), the viability test in chondrocytes T/C-28a2 allowed us to use pathological FA concentrations concerning normal serum levels (Joosten et al., 2010; Zhang et al., 2014).

In summary, based on the results obtained in our study, saturated FA, such as PA, produce harmful effects, a mitochondrial dysfunction as well as alterations in glycolytic metabolism. These events can trigger OA pathology through the degradation of the MEC of articular cartilage. Importantly, these effects can be partially reversed by monounsaturated FA, such as OL. These results may help explain, at least in part, the close relationship of metabolic pathologies with OA, as well as help to elucidate some of the factors that can define a metabolic phenotype in OA. Accurate identification of this phenotype would lead to the specific treatment that helps to combat metabolic disorders in these patients, preventing, in turn, the appearance as well as the OA evolution.

## Data Availability Statement

The raw data supporting the conclusions of this article will be made available by the authors, without undue reservation.

## Author Contributions

FB and IR-P contributed equally in the design and coordination of the study, conceived the study, participated in its design, and helped to draft the final version of the manuscript. MV-M carried out the experiments and helped to draft the manuscript and data interpretation. MF-M supervised and helped to develop the experimental of mitochondrial and glycolytic function and interpretation. EC-P helped to carry out the chondrocytes culture. SR helped to carried out the stains and cytometry experiments. AD-F, AD, and PR-L helped to carry out the RNA isolation and real-time PCR experiments. All authors approved the final version of the manuscript.

## Conflict of Interest

The authors declare that the research was conducted in the absence of any commercial or financial relationships that could be construed as a potential conflict of interest.
