# Supplementary material for: Oleate Prevents Palmitate-Induced Mitochondrial Dysfunction in Chondrocytes
Source: Front Physiol. 2021 Jun 15;12:670753. doi: 10.3389/fphys.2021.670753 (PMC8239231; doi:10.3389/fphys.2021.670753)
Supplement: Supplementary file 1 [file Data_Sheet_1.PDF]

Oleate prevents palmitate-induced mitochondrial dysfunction in chondrocytes

Vázquez-Mosquera ME[1], Fernández-Moreno M[1], Cortés-Pereira E[1], Relaño S[1], Dalmao-Fernández A[1], Durán-Sotuela A[1], Ramos-Louro P[1],Rego-Pérez I[1*], Blanco FJ[1*]

1) Servicio de Reumatología. Instituto de Investigación Biomédica de A Coruña (INIBIC). Complexo Hospitalario Universitario de A Coruña (CHUAC), Sergas. Universidade da Coruña (UDC), Spain.

* Corresponding authors: Fblagar@sergas.es and Ignacio.rego.perez@sergas.es

Table S1. UPL primers and probes of the analyzed genes grouped by their function

FUNCTION	GENE	PROTEIN	P. SENSE (5´-3´)<br>P.ANTISENSE (5´-3´)	UPL PROBE
Metabolism	**SLC2A3**	Solute carrier family 2 member 3	gggtgtggttaatactatcttcactg	# 31
			cccagtctctccacgttcac	
	**HK2**	Hexokinase 2	agaagctcccactgggtttt	# 87
			catgagaccaggaaactctcg	
			tcagatgtgtctgaggatttctct	
Reference gene	**RPL13A**	Ribosomal protein L13a	caagcggatgaacaccaac	# 28
			tgtggggcagcatacctc	

Table S2. Parameters calculated to assess mitochondrial function from the oxygen consumption rate (OCR) **(a)** and glycolytic function from the extracellular acidification rate (ECAR) **(b)**

(a)

Parameter	Description
**Basal respiration**	Baseline respiration before addition of compounds
**ATP synthesis**	**Oligomycin**-sensitive respiration: basal respiration portion used for ATP production
**Proton leak**	**Oligomycin**-insensitive respiration: represents remaining basal respiration not coupled to ATP production
**Coupling efficiency**	Ratio of ATP turnover-linked respiration and basal respiration: an indicator of OXPHOS and electron transport chain coupling quality
**Maximal respiration**	After the addition of an uncoupler (**FCCP**): reflects the maximal capacity of the electron transport chain
**Spare respiratory capacity**	Difference between the maximum respiration obtained after the addition of FCCP and basal respiration: indicates the ability of the cell to respond to an energy demand
**Non-mitochondrial respiration**	Rate is also necessary to obtain an accurate measurement of the above parameters. This is represented by the final OCR after the addition of **Rot/AntA**

(b)

Parameter	Description
**Glycolysis rate**	Represents the process of converting glucose into pyruvate: It is measured as the rate of ECAR reached by the cells after **Glucose** addition
**Glycolytic capacity**	Maximum rate of ECAR reached after the addition of **Olygomicin** that inhibits OXPHOS, forcing the cells to use glycolysis at maximum capacity
**Glycolytic reserve**	Difference between the maximum glycolytic capacity and glycolysis: indicates the cells' ability to respond to an energy demand

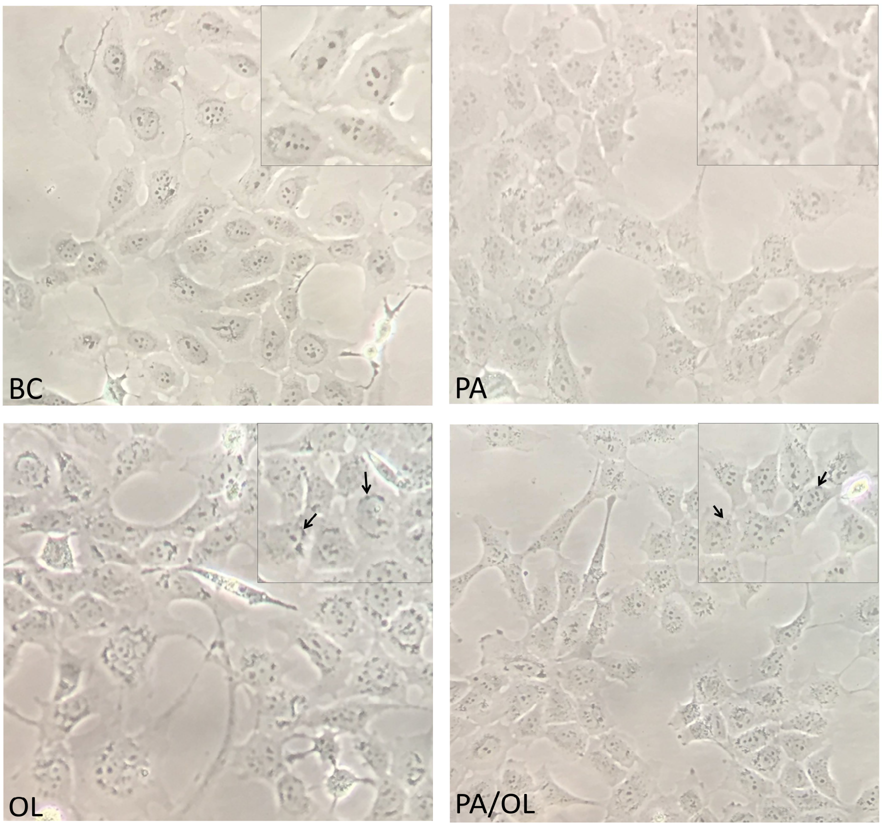

Figure S1. Representative images of the structural changes detected in chondrocytes by phase contrast optical microscopy (200x). BC:basal condition; PA: palmitic acid; OL:oleic acid.
